# Systemic Immune Profiling Reveals Candidate Biomarkers in Luminal A Breast Cancer: A Comparative Pilot Study

**DOI:** 10.3390/biomedicines13112787

**Published:** 2025-11-14

**Authors:** Tânia Moura, Olga Caramelo, Isabel Silva, Sandra Silva, Paula Laranjeira, Artur Paiva

**Affiliations:** 1 Flow Cytometry Unit, Department of Clinical Pathology, Hospitais da Universidade de Coimbra, Unidade Local de Saúde de Coimbra, 3000-076 Coimbra, Portugal; tania.moura@ua.pt (T.M.); 14546@ulscoimbra.min-saude.pt (I.S.); 9656@ulscoimbra.min-saude.pt (S.S.); 1979paula@gmail.com (P.L.); 2 Gynecology Department, Hospitais da Universidade de Coimbra, Unidade Local de Saúde de Coimbra, 3000-075 Coimbra, Portugal; olgalgcaramelo@gmail.com; 3 Group of Environmental Genetics of Oncobiology (CIMAGO), Coimbra Institute for Clinical and Biomedical Research (iCBR), Faculty of Medicine (FMUC), University of Coimbra, 3000-548 Coimbra, Portugal; 4 Center for Innovative Biomedicine and Biotechnology (CIBB), University of Coimbra, 3000-504 Coimbra, Portugal; 5 Clinical Academic Center of Coimbra (CACC), 3000-061 Coimbra, Portugal; 6 Center of Neurosciences and Cell (CNC), University of Coimbra, 3000-504 Coimbra, Portugal; 7 Ciências Biomédicas Laboratoriais, Instituto Politécnico de Coimbra, ESTESC—Escola Superior de Tecnologia da Saude de Coimbra, Coimbra Health School, 3046-854 Coimbra, Portugal; 8 Centro de Inovação e Desenvolvimento, Unidade Local de Saúde de Coimbra, 3004-561 Coimbra, Portugal

**Keywords:** luminal A breast cancer, peripheral immune profile, flow cytometry, biomarkers

## Abstract

**Background:** Luminal A breast cancer, the most common molecular subtype, is typically associated with a favorable prognosis. However, its systemic immune landscape remains largely uncharacterized. **Methods:** In this study, we used high-dimensional flow cytometry to characterize peripheral immune alterations in 13 patients with luminal A breast cancer compared to 14 age-matched healthy female controls. A total of 254 immune subsets were analyzed, including 23 innate populations and 231 T cell subpopulations, defined by detailed phenotypic and functional markers. **Results:** The main observations in the luminal A breast cancer group included a significant increase in neutrophils, plasmacytoid dendritic cells (pDCs), and CD4^+^ follicular T lymphocytes, as well as a reduced percentage of monocytes, conventional type 2 dendritic cells (cDC2), and CD4^+^CD196^+^ T cells. **Conclusions:** Despite being a preliminary study, these findings highlight distinct immune alterations in luminal A breast cancer and support the use of flow cytometry for identifying biomarkers, measurable biological indicators of disease presence, progression, or therapeutic response.

## 1. Introduction

Breast cancer is the most commonly diagnosed malignancy and the leading cause of cancer-related mortality among women worldwide, exhibiting significant clinical, molecular and therapeutic heterogeneity [1,2]. Among its subtypes, luminal A breast cancer is the most prevalent, accounting for approximately 60–75% of all cases. It is characterized by hormone receptor positivity, a low proliferative index and reduced Ki-67 expression, generally associated with a favorable prognosis [3,4].

Despite its generally indolent behavior, growing evidence suggests that immune dysregulation in luminal A breast cancer may influence tumor progression, recurrence, and treatment response [5,6]. While immune alterations have been extensively studied in triple-negative and HER2+ subtypes, the systemic immune landscape of luminal A breast cancer remains largely underexplored [7,8].

Circulating immune cells, including monocytes, dendritic cells, and T cells, play essential roles in anti-tumor immunity and are frequently reprogrammed in the cancer context [9,10,11]. Additionally, estrogen modulates immune responses, promoting Treg expansion and suppressing cytotoxicity [12,13].

In this study, high-dimensional flow cytometry was employed to comprehensively profile circulating immune cells in luminal A breast cancer, aiming to identify immune alterations of potential clinical relevance.

## 2. Materials and Methods

### 2.1. Participants and Sample Collection

Peripheral blood samples were obtained from 13 patients with luminal A breast cancer and 14 age-matched healthy controls, collected in EDTA tubes either on the day of surgery or the day before, and processed at the Flow Cytometry Unit, Unidade Local de Saúde de Coimbra, Portugal. All participants provided written informed consent, and the study was approved by the Ethics Committee (Ref.: 017/24 CE). Clinical and pathological data are detailed in Table 1. Of all patients, 92.3% underwent radiotherapy and were under hormonal therapy at the time of flow cytometry analysis.

### 2.2. Characterization of Immune Cells by Flow Cytometry

Peripheral blood samples (200 µL) were incubated with monoclonal antibodies (Supplementary Table S1) for 10 min at room temperature (RT) in the dark. Then, 2 mL of FACSLysing Solution (BD Biosciences (BD), San Jose, CA, USA) was added, followed by another 10 min incubation under the same conditions. After centrifugation (540× *g*, 5 min), the supernatant was discarded, and the cell pellet was washed with 2 mL of PBS. The final cell suspension was prepared in 500 µL of PBS for acquisition on a FACSLyric flow cytometer (BD) using FACSuite software (v.1.5.0925; BD). Data acquisition was performed using standardized instrument settings recommended by the EuroFlow consortium.

### 2.3. Flow Cytometry Analysis

Peripheral immune cell populations were analyzed by flow cytometry, as detailed in Supplementary Methods.

### 2.4. Statistical Analysis

Statistical analyses were performed using GraphPad Prism 8 (GraphPad Software Inc., La Jolla, CA, USA), with normality assessed by Shapiro–Wilk test. Parametric data were analyzed using Student’s *t*-test, and non-parametric data were evaluated using the Mann–Whitney test. A *p* < 0.05 was considered statistically significant. Results are shown as mean ± standard deviation (SD). ROC analyses were performed using MedCalc (v23.2.1; MedCalc Software Ltd., Ostend, Belgium).

## 3. Results

### 3.1. Cellularity Characterization in Samples from Patients with Luminal A Breast Cancer

**Figure 1:**
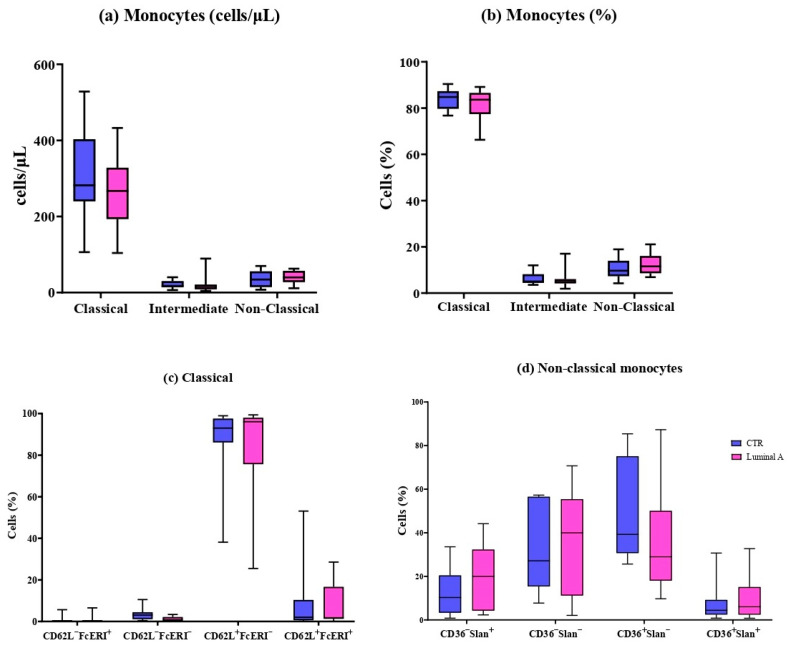
Peripheral blood monocyte subsets in control (blue) and luminal A patients (pink). (**a**) Absolute counts (cells/μL) and (**b**) percentages of classical, intermediate, and non-classical monocytes. (**c**) CD62L and FcεRI expression in classical monocytes. (**d**) CD62L, FcεRI, and HLA-DR expression in non-classical monocytes. Boxes show median and interquartile range.

The analysis of peripheral blood cells in luminal A breast cancer revealed significant immune alterations compared with controls. Although total white blood cell counts were slightly higher, the difference was not statistically significant (*p* > 0.05) (Figure 1 and Table 2). In contrast, neutrophil counts significantly increased (*p* < 0.05), whereas monocytes and dendritic cell percentages were significantly decreased (*p* < 0.05) (Table 2). No significant differences were observed in the absolute number of total dendritic cells between groups (*p* > 0.05), but their relative frequency was lower in the luminal A group (*p* < 0.05). Immune profiles observed in the control group were consistent with reference values previously reported using EuroFlow PID Orientation Tube [15].

### 3.2. Detailed Immunophenotyping of 11 Monocyte and 6 Dendritic Cell Subpopulations

Seventeen immune cell subpopulations were analyzed, including eleven monocyte subsets and six dendritic cell subsets. In both luminal A breast cancer patients and controls, classical monocytes were identified as the dominant population. No significant differences were observed among the classical, intermediate, and non-classical subsets (Figure 1). Similarly, the expression-based subtypes defined by markers such as CD36, Slan, CD62L, and FcεRI were not found to differ significantly between the two groups.

The cDC1 subpopulation did not differ significantly in either absolute number or frequency (*p* > 0.05) (Table 3). In contrast, pDCs were significantly increased in both absolute number and percentage in the luminal A group, whereas cDC2 cells were significantly decreased in both measures (*p* < 0.001) (Table 3).

**Figure 2:**
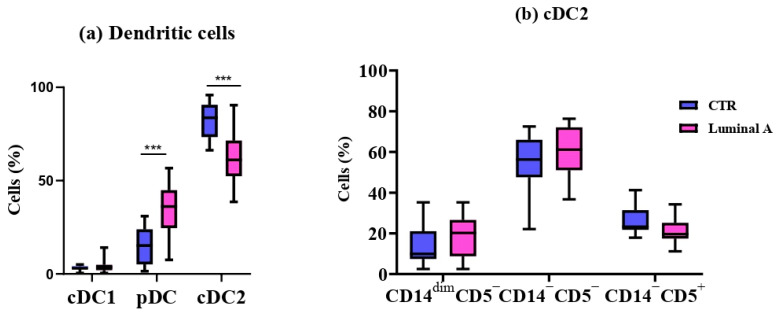
Comparison of dendritic cell subsets between controls (CTR) and Luminal A patients. (**a**) Frequencies of cDC1, pDC, and cDC2 in peripheral blood. (**b**) Phenotypic profile of cDC2 based on CD15, CD5, and CD14 expression. Boxes show median and interquartile range. Statistical analysis was performed using the Mann–Whitney test. *** *p* < 0.001 was considered significant.

No statistically significant differences were observed in cDC2 cell subpopulations between the control and luminal A groups (*p* > 0.05) (Figure 2).

### 3.3. Extensive Immunophenotypic Characterization of 234 T Cell Subsets

T cell immune profiling revealed no significant differences (*p* > 0.05) between the luminal A patients and controls in the frequencies or absolute counts of the major subpopulations, including CD4^+^, CD8^+^, γδ T, and CD4^−^CD8^−^ cells (Table 4).

Notably, although not statistically significant, a discernible trend was observed toward increased levels of CD4^+^CD8^+^ T cells in the luminal A group. Immune profiles in the control’s individuals were consistent with reference ranges established by the Euroflow PID Orientation Tube [15], supporting the robustness of the cytometry analysis applied.

The activation profile of T cell subpopulations was analyzed and showed limited differences between luminal A and control groups, as assessed by CD25 and HLA-DR expression across major T cell subpopulations (Table 5). While most comparisons did not reach statistical significance, a prominent increase in CD4^+^CD25^+^ T cells was observed in the luminal A group (*p* < 0.05).

#### Analysis of T Cell Subpopulations in CD4^+^ and CD8^+^ Cells in Control and Luminal A Breast Cancer

**Figure 3:**
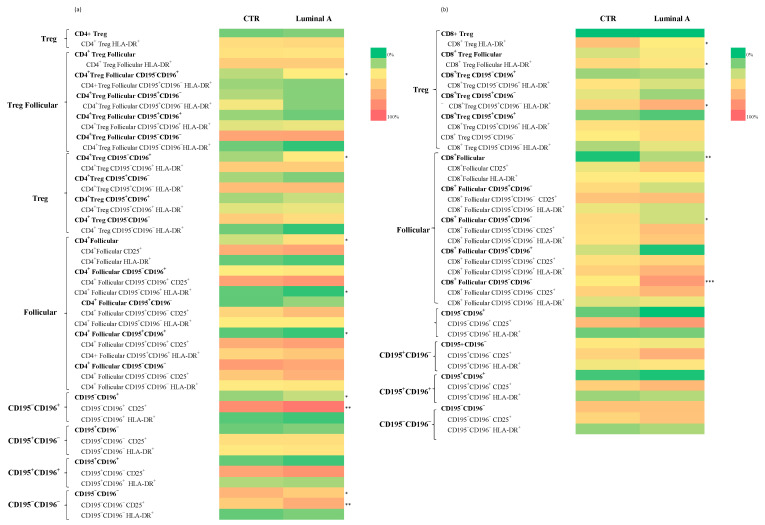
Analysis of T cell subpopulations in control and luminal A samples revealed differences in the distribution and activation status of CD4^+^ (**a**) and CD8^+^ (**b**) T cell subsets. Statistically significant differences are indicated as *** *p* < 0.001, ** *p* < 0.01 and * *p* < 0.05.

A comparison between control samples and patients with luminal A breast cancer revealed significant phenotypic alterations in CD4^+^ and CD8^+^ T cell subsets. Within the CD4^+^ T cells, statistically significant decreases were observed in the frequency of CD4^+^ Treg follicular T cells CD195^−^CD196^+^ (*p* < 0.05) and CD4^+^ Treg CD195^−^CD196^+^ (*p* < 0.05) in the Luminal A group (Figure 3 and Supplementary Table S4). In contrast, increased frequencies of CD4^+^ follicular cells expressing CD195^+^CD196^+^ (*p* < 0.05) and CD195^−^CD196^+^ CD25^+^ (*p* < 0.01) were detected (Supplementary Tables S4 and S5). Furthermore, CD195^−^CD196^−^ and CD195^−^CD196^−^ CD25^+^ subsets were significantly reduced (Figure 3 and Supplementary Table S2) (*p* < 0.05 and *p* < 0.01, respectively).

In the CD8^+^ T cells, significant increases were observed in the frequencies of CD8^+^ Treg HLA-DR^+^ (*p* < 0.05) and CD8^+^ Treg follicular HLA-DR^+^ cells (*p* < 0.05) in Luminal A patients (Figure 3 and Supplementary Tables S3 and S5). Additionally, CD8^+^ follicular CD195^+^CD196^−^ (*p* < 0.05), CD8^+^ follicular CD195^−^CD196^−^ (*p* < 0.001), and CD195^−^CD196^−^ CD25^+^ (*p* < 0.01) subsets were found to be significantly increased. CD8^+^ Treg CD195^+^CD196^−^ HLA-DR^+^ cells also showed increased frequencies (*p* < 0.05) (Supplementary Table S5).

**Figure 4:**
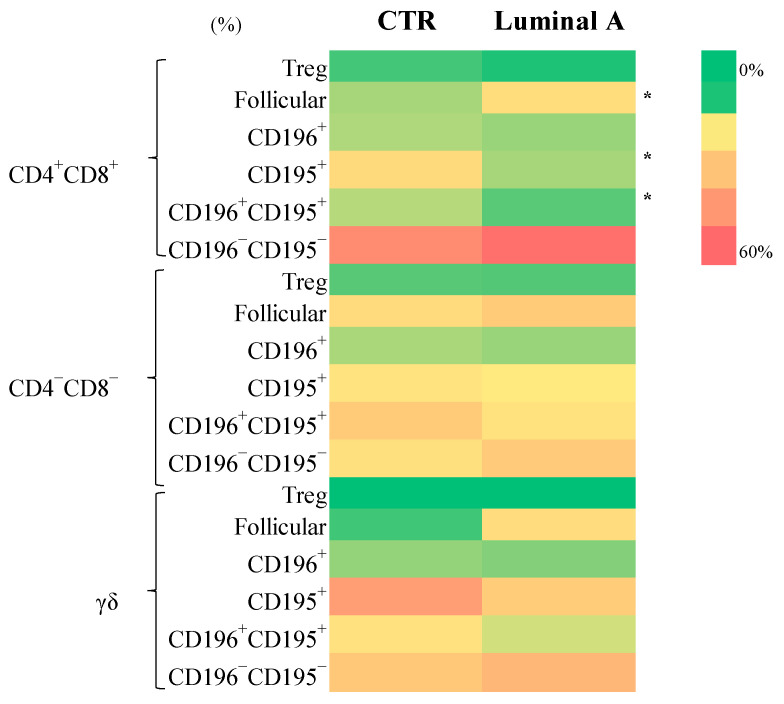
Frequency of CD4^+^CD8^+^, CD4^−^CD8^−^, and γδ T cell subsets in control (CTR) and luminal A groups. Statistically significant differences are indicated as * *p* < 0.05.

Subpopulations within the CD4^+^CD8^+^, CD4^−^CD8^−^, and γδ T cell compartments were analyzed to assess their distribution of luminal A breast cancer patients. Within the CD4^+^CD8^+^ subset, follicular cells, as well as CD195^+^, and CD195^+^CD196^+^ populations, were significantly increased in luminal A patients (*p* < 0.05) (Figure 4, Supplementary Table S6). Similarly, γδ follicular T cells were elevated in luminal A (*p* < 0.05), while no significant differences were observed in other γδ T cell subsets.

Regarding activation markers, luminal A patients exhibited a significant decrease in CD4^+^CD8^+^HLA-DR^+^ regulatory T cells (*p* < 0.05). Conversely, the CD4^−^CD8^−^CD25^+^ subset displayed significant increases in CD196^+^, CD195^+^CD196^+^, and CD195^−^CD196^−^ populations (*p* < 0.001 to *p* < 0.05) in luminal A patients. No significant differences were found in CD4^−^CD8^−^HLA-DR^+^, γδ T HLA-DR^+^, or γδ T CD25^+^ subpopulations between groups (Supplementary Table S6).

**Figure 5:**
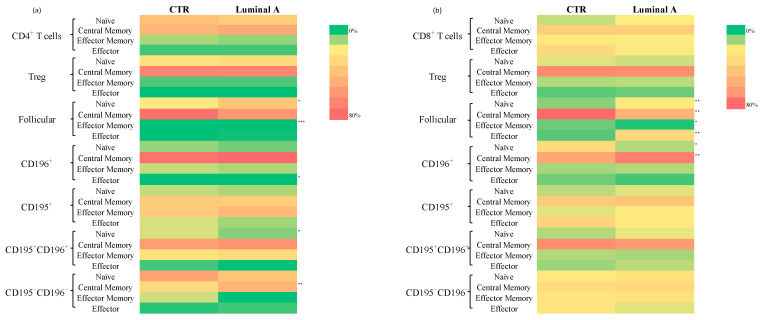
Heatmap represents the distribution of T cell memory compartments in controls and Luminal A breast cancer patients. Statistically significant differences are indicated as *** *p* < 0.001, ** *p* < 0.01 and * *p* < 0.05. (**a**) relative distribution of naïve, central memory, effector memory and effector cells in CD4^+^ T cell subsets; (**b**) relative distribution of naïve, central memory, effector memory and effector cells in CD8^+^ T cell subsets.

Further characterization of the memory phenotype revealed a higher proportion of central memory CD4^+^ T cells in the luminal A group (Figure 5 and Supplementary Table S8). Among CD4^−^CD8^−^ T cells, both naïve and effector cells were increased, whereas central memory cells were reduced, compared to controls (Supplementary Table S8). No other significant differences were noted across the remaining T cell subsets.

Analysis of T cell maturation showed a consistent increase in naïve and terminal effector cells, accompanied by a reduction in central and effector memory subsets across CD4^+^ and CD8^+^ follicular T cells (*p* < 0.01). Notably, Tregs follicular CD195^+^ cells were diminished within the central memory compartment, whereas naïve Tregs CD195^−^CD196^−^ follicular cells were enriched (*p* < 0.05) (Supplementary Table S9).

### 3.4. Diagnostic Performance of Biomarkers in the Classification of Luminal A Breast Cancer

The analysis showed that pDC and cDC2 had the highest accuracy in distinguishing luminal A breast cancer patients from controls, demonstrating excellent classification performance for identifying positive cases. CD4^+^CD195^−^CD196^+^ cells were found to exhibit high sensitivity but lower specificity, resulting in an increased rate of false positives.

In addition to dendritic cells, CD4^+^CD8^+^CD195^+^ and CD8^+^ follicular CD195^−^CD196^−^ cells showed high diagnostic performance, with AUC values comparable to those of pDCs and cDC2. CD4^+^CD195^−^CD196^+^ and CD8^+^ follicular cells also demonstrated good results, though with lower specificity (Table 6).

## 4. Discussion

Emerging evidence underscores the critical role of the immune system in breast cancer development and progression. Although luminal A is the most frequent and clinically less aggressive subtype, characterized by low proliferation, estrogen receptor positivity, and favorable prognosis, it remains unclear whether systemic immune alterations are present and contribute to disease progression. In fact, immune dysregulation has been more extensively explored in triple negative and HER2+ breast cancer, while luminal A has received comparatively less attention [3,16].

In this study, a total of 70 statistically significant differences were identified between patients with luminal A breast cancer and healthy controls. Of these, 5 involved innate immune populations, while 65 referred to alterations within T cells subsets.

Within the innate immune compartment, luminal A patients exhibited increased circulating neutrophils, contributing to a shift toward a higher neutrophils-to-lymphocyte ratio. Notably, recent findings suggest that even within the luminal A subtype, typically associated with better outcomes, an elevated neutrophil-to-lymphocyte ratio may reflect systemic inflammation and immune dysregulation linked to poorer prognosis. This immune profile may be further modulated by estrogen signaling pathways, which have been shown to influence both neutrophil expansion and lymphocyte suppression [17,18,19]. These observations underscore the relevance of immune–endocrine interactions in shaping tumor behavior and highlight the potential of neutrophil-to-lymphocyte ratio as a complementary marker in subtype-specific breast cancer risk stratification.

Importantly, when interpreting systemic immune alterations in luminal A breast cancer, treatment-related factors must be considered. Radiotherapy and endocrine therapy are known to modulate immune responses in cancer patients. Radiotherapy can induce transient or persistent lymphopenia, alter the distribution of T cell subsets, and promote the release of inflammatory cytokines [20]. Similarly, endocrine therapy, particularly involving selective estrogen receptor modulators or aromatase inhibitors, has been shown to influence cytokine production and immune cell activity [21]. These therapeutic interventions may contribute to some of the immune differences observed between patients and healthy controls. However, given the consistency of therapeutic regimens across the patient cohort, the comparative analysis remains valid and reflective of the clinical context.

Beyond treatment-related factors, several intrinsic immune alterations were also evident. A reduction in circulating monocytes was observed, potentially reflecting their recruitment to the tumor microenvironment or functional exhaustion [22,23,24]. Additionally, we identified a marked reduction in DCs in the luminal A group, particularly a decrease in plasmacytoid dendritic cells accompanied by an increase in cDC2. Interestingly, this altered pDC number contrasts with reports in HER2+ breast cancer, where an increase in pDCs was observed, highlighting breast cancer subtype-specific immune dynamics [25]. Of note, pDC and cDC2 demonstrated the highest diagnostic performance in ROC analysis, outperforming other immune cell populations in their ability to discriminate between luminal A patients and healthy controls.

Regarding adaptive immunity, frequencies of major T cell subsets were overall comparable between Luminal A and control groups. Nevertheless, a trend towards increased circulating CD4^+^CD8^+^ T cells was observed in luminal A patients. Although these cells are typically rare in peripheral blood, their expansion has been reported in chronic inflammatory conditions and cancer, where they exhibit dual helper and cytotoxic functions [19,26,27].

Concerning Tregs, no significant increase was observed in total circulating Treg population in luminal A compared to controls. However, a significant increase was detected in the Treg follicular subset. Treg follicular cells play a crucial role in controlling excessive B cell activation and maintaining immune tolerance within germinal centers. This increase has been described in specific breast cancer subtypes [28,29].

We also observed a reduction in follicular T cells, which are essential for B cell activation and subsequent antibody production, which could be due to estrogen-mediated suppression of follicular T cell differentiation [30].

Among functional T cell populations, CD195^−^CD196^+^ cells, described in the literature as Th17 cells, were increased in luminal A patients. Th17 cells are known to secrete IL-17 and are involved in chronic inflammation, angiogenesis, and tumor progression, although their role in cancer remains controversial [31].

## 5. Conclusions

Recent flow cytometry approaches allow a deeper evaluation of the peripheral immune system and characterization of multiple immune cells subpopulations. These advances are likely to impact cancer progression and biomarker discovery. This study is a good example of this, as it reveals significant systemic immune alterations in patients with luminal A breast cancer. Together, these findings reveal an altered immune profile in luminal A breast cancer, marked by neutrophilic inflammation, dendritic cell depletion, expansion of regulatory T cells and Th17 cells, and impaired follicular T cell function.

The obtained results, once confirmed in a larger patient cohort, could be integrated into algorithms for the identification of clinically relevant biomarkers in breast cancer subtypes and contribute to personalized treatment strategies, particularly in combination with endocrine therapy.

## Figures and Tables

**Table 1:** Demographic and clinical characteristics of breast cancer patients. Tumor staging was performed according to the 8th edition American Joint Committee on Cancer (AJCC) Cancer Staging Manual [14].

Variable	*n* = 13
Age	
Mean ± SD	63 ± 7
**Histological Grade, *n* (%)**	
I	10 (77%)
II	3 (23%)
III	0 (0%)
**Ki-67, *n* (%)**	
>5	8 (67%)
5–10	3 (25%)
10–15	1 (8%)
**Tumor Size, *n* (%)**	
5–10 mm	3 (23%)
10–20 mm	8 (62%)
<20 mm	2 (15%)
**Lymph Node Status, *n* (%)**	
N0	10 (77%)
N1	3 (23%)
N2	0 (0%)

**Table 2:** Peripheral blood leukocyte populations in control individuals (CTR) and patients with luminal A breast cancer, shown as absolute counts (cells/μL) and percentages (%).

Cells Type	Controls	Luminal A	*p*-Value
WBC (cells/μL)	4914 ± 1492	6012 ± 1450	0.06
**Eosinophils (cells/μL)**	116 ± 95	78 ± 51	0.22
Eosinophils (%)	2.25 ± 1.59	1.41 ± 1.12	0.09
**Neutrophils (cells/μL)**	2881 ± 1188	3679 ± 1070 *	0.05
Neutrophils (%)	58 ± 9.78	62 ± 11	0.37
**Monocytes (cells/μL)**	354 ± 137	328 ± 122	0.49
Monocytes (%)	7.21 ± 2.08	5.67 ± 2.64 *	0.034
**Lymphocytes (cells/μL)**	1512 ± 585	1843 ± 895	0.27
Lymphocytes (%)	32 ± 9.37	31 ± 11.7	0.63
**T cells (cells/μL)**	1031 ± 615	1204 ± 629	0.40
T cells (%) (within lymphocytes)	21 ± 10	20 ± 10	0.65
**Dendritic cells (cells/μL)**	16 ± 6.19	14 ± 5.88	0.53
Dendritic cells (%)	0.33 ± 0.10	0.24 ± 0.12 *	0.029
**Basophils (cells/μL)**	36 ± 27	35 ± 19	0.85
Basophils (%)	0.74 ± 0.44	0.57 ± 0.25	0.24

All data are shown as mean ± SD. Statistical analysis was performed using the Mann–Whitney test. * *p* < 0.05 was considered significant.

**Table 3:** Comparison of dendritic cell subtypes between controls and Luminal A breast cancer patients.

Dendritic Cell Subtypes	Controls	Luminal A	*p*-Value
**cDC1 (cells/uL)**	0.52 ± 0.32	0.71 ± 0.88	0.78
cDC1 (%)	0.0105 ± 0.005	0.012 ± 0.0156	0.374
**pDC (cells/uL)**	2.31 ± 1.85	4.88 ± 2.94 *	0.017
pDC (%)	0.049 ± 0.037	0.085 ± 0.056	0.095
**cDC2 (cells/uL)**	13 ± 5.64	8.49 ± 3.15 *	0.04
cDC2 (%)	0.26 ± 0.088	0.146 ± 0.065 ***	0.0001

All data are shown as mean ± SD. Statistical analysis was performed using the Mann–Whitney test. * *p* < 0.05 was considered significant, *** indicates *p* < 0.001.

**Table 4:** Analys of principal T cell subpopulations in control and luminal A groups.

T Cells	Controls	Luminal A	*p*-Value
**CD4^+^ (cells/μL)**	499 ± 192	630 ± 342	0.26
CD4^+^ (%)	61 ± 12	64 ± 11	0.54
**CD8^+^ (cells/μL)**	251 ± 163	341 ± 291	0.35
CD8^+^ (%)	33 ± 12	30 ± 9.46	0.40
**CD4^+^CD8^+^ (cells/μL)**	9.05 ± 4.77	24 ± 30	0.10
CD4^+^CD8^+^ (%)	1.41 ± 1.21	2.70 ± 3.16	0.16
**CD4^−^CD8^−^ (cells/μL)**	3.42 ± 2.84	5.82 ± 5.16	0.17
CD4^−^CD8^−^ (%)	0.59 ± 0.33	0.62 ± 0.20	0.49
**γ** **δ (cells/** **μL)**	24 ± 21	27 ± 30	0.77
γδ (%)	3.31± 3.25	2.57 ± 3.04	0.45

All data are shown as mean ± SD. Statistical analysis was performed using the Mann–Whitney test.

**Table 5:** Frequency of activated T cells subpopulations in control and Luminal A groups.

T Cells	Controls	Luminal A	*p*-Value
**CD4^+^ CD25^+^**	49 ± 11	67 ± 12 **	0.001
**CD4^+^ HLA-DR^+^**	9.14 ± 6.98	8.11 ± 2.6	0.87
**CD8^+^ CD25^+^**	39 ± 39	59 ± 40	0.21
**CD8^+^ HLA-DR^+^**	20 ± 9.47	16 ± 11	0.23
**CD4^+^CD8^+^ CD25^+^**	44 ± 27	51 ± 30	0.48
**CD4^+^CD8^+^ HLA-DR^+^**	17 ± 12	12 ± 7.73	0.16
**CD4^−^CD8^−^ CD25^+^**	24 ± 13	23 ± 6.13	0.99
**CD4^−^CD8^−^ HLA-DR^+^**	33 ± 19	33 ± 15	0.99
**γ** **δ CD25^+^**	19 ± 17	19 ± 14	0.93

All data are shown as mean ± SD. Statistical analysis was performed using the Mann–Whitney test. ** *p* < 0.01 was considered significant.

**Table 6:** Diagnostic performance of selected biomarkers, including cut-off values, sensitivity, specificity, AUC, and *p*-values.

Biomarker	Threshold	Sensitivity	Specificity	AUC	*p*-Value
cDC2	0.69	69	100	0.86	<0.0001
pDC	0.69	69	100	0.87	<0.0001
CD4^+^ Follicular	20	62	93	0.77	0.015
CD4^+^ Treg CD195^−^CD196^+^	12.28	57	92	0.79	0.012
CD4^+^CD8^+^ CD195^+^	11.94	77	85	0.81	0.006
CD8^+^ Follicular	2.55	85	64	0.80	0.007
CD8^+^ Follicular CD195^−^CD196^−^	29	85	62	0.87	0.0014
CD8^+^ Follicular	2.41	85	57	0.80	0.007

## Data Availability

Data available on request from the authors.

## Supplementary Materials

The following supporting information can be downloaded at: https://www.mdpi.com/article/10.3390/biomedicines13112787/s1, Figure S1: Detailed hierarchy of phenotypic classification of leukocytes and CD4^+^ T cell subpopulations; Table S1: Panel of monoclonal antibodies used for the characterization of immune cells, indicating their respective fluorochrome, clone and commercial source; Table S2: Comparison of T cell subpopulations across maturation compartments between the control group and Luminal A group; Table S3: Analys of principal T cell subpopulations in control and luminal A groups; Table S4: Frequency of activated T cell subsets in controls and Luminal A. Activation was assessed by CD25 and HLA-DR expression in different T cell populations; Table S5: Maturation compartments of CD4^+^ and CD8^+^ T cells in the control and Luminal A groups. The data show the distribution of T cell subsets, including naïve, central memory, effector memory, and terminal effector cells, across different populations such as Treg, follicular, Th17, Th1, Th17/1, and CD195^−^CD196^−^; Table S6: Phenotypic characterization of CD4^+^ and CD8^+^ T cell subsets in peripheral blood from healthy controls and luminal A breast cancer patients; Table S7: Activation profile of CD4^+^ and CD8^+^ T cell subsets in peripheral blood from healthy controls and luminal A breast cancer patients; Table S8: Maturation-associated distribution of follicular CD4^+^ and CD8^+^ T cell subsets in peripheral blood from healthy controls and luminal A breast cancer patients; Table S9: Phenotypic and activation profiling of CD4^+^CD8^+^, CD4^−^CD8^−^ and γδ T cell subsets in peripheral blood from healthy controls and luminal A breast cancer patients.
