# Supplementary material for: Systemic Immune Profiling Reveals Candidate Biomarkers in Luminal A Breast Cancer: A Comparative Pilot Study
Source: Biomedicines. 2025 Nov 14;13(11):2787. doi: 10.3390/biomedicines13112787 (PMC12650388; doi:10.3390/biomedicines13112787)
Supplement: Supplementary file 1 [file biomedicines-13-02787-s001.zip › biomedicines-3970337-supplementary.pdf]

Table S1 – Panel of monoclonal antibodies used for the characterization of immune cells, indicating their respective fluorochrome, clone and commercial source.

**Fluorochrome**	**FITC**	**PE**	**PerCPCy5.5**	**PE-Cy7**	**APC**	**APC-R700**	**APC-H7**	**PB**	**PO**	**BV605**	**BV711**	**BV786**
**Tube 1**												
**Antibody marker**	**CD1c**	**Slan + FcEri**	**CD36**	**CD33**	**CD300e+CD303**	**CD45**	**CD14**	**CD141**	**CD5**	**CD62L**	**HLA-DR**	**CD16**
**Clone**	F10/21[a]3	DD1 + ERA-37 (CRA1)	CLB-IVC7	P67.6	UP-H2 + AC144	HI30	MoP9	1A4	UCHT2	DREG-56	G46-6	3G8
**Commercial source**	BD	Miltenyi + Thermo Fisher	BD	BD	Immunostep + Miltenyi	BD	bd	BD	BD	Biolegend	BD	BD
**Tube 2**												
**Antibody marker**	TCRγδ	**CD4**	**CD196**	**CD127**	**CD25**	**CD27**	**CD8**	**CD185**	**HLA-DR**	**CD3**	**CD195**	**CD45RA**
**Clone**	IMMU510	SK3	11A9	HIL-7R-M21	2A3	M-T271	SK1	RF8B2	G46-6	SK7	2D77CCR5	HI100
**Commercial source**	Beckman Coulter	BD	BD Pharmingen	BD Pharmingen	BD	BD Horizon	BD	BD Horizon	BD Horizon	BD Horizon	BD Horizon	BD Horizon

Abbreviations: APC—Allophycocyanin; APC-H7—Allophycocyanin-hilite 7; BV—Brilliant violet; FITC—Fluorescein isothiocyanate; PB—Pacific blue; PE—Phycoerythrin; PerCP-Cy5.5—Peridinin chlorophyll protein cyanine 5.5; PE-Cy7—Phycoerythrin Cyanine 7; PO—Pacific orange. Commercial source: BD—Becton Dickinson Biosciences, San Jose, CA, USA; BD Horizon—Franklin Lakes, NJ, USA; BD Pharmingen, San Diego, CA, USA; Beckman Coulter—Miami, FL, USA; Biolegend—San Diego, CA, USA.

Flow cytometry analysis

Data were analyzed using Infinicyt™ software (V.2.05; Cytognos SL, Salamanca, Spain). Immune cells were identified by CD45 expression and characteristic immunophenotyping profiles. Granulocytes were distinguished by their FSC/SSC properties and CD16 expression, with neutrophils defined as $CD16^+$, and eosinophils as $CD16^-$. Monocytes ($CD33^+$) were classified into classical ($CD14^+CD16^-$), intermediate ($CD14^+CD16^+$), and non-classical ($CD14^-CD16^-$), with further stratification of non-classical subsets by Slan and CD36, and classical monocytes by CD62L and FcεRI.

Dendritic cells were classified as pDCs ($CD303^+$, $HLA\text{-}DR^+$, $CD14^-CD16^-$), cDC1 ($CD141^{dim}/^-$, $Fc\varepsilon RI^+$) with three subsets (CD5+CD14dim, CD5+CD14-, CD14-CD5-) and cDC2 ($CD141^+$, $CD300e^+$, $HLA\text{-}DR^+$, $CD303^-$). Basophils were identified as $CD33^+$, $SLAN^+Fc\varepsilon RI^+CD62L^+$, and negative for CD14, HLA-DR, and CD300e. Lymphocytes were gated as CD45 high and low granularity.

T cells were identified based on CD3 positivity. Subsets of T cells were further classified as follows: CD4+ T cells (defined by the presence of CD4 and absence of CD8), $CD8^+$ T cells (expressing CD8 but lacking CD4), double-positive CD4+CD8+ T cells (co-expressing both markers), double-negative $CD4^-CD8^-$ T cells (with a $CD3^+CD4^-CD8^-\gamma\delta^-$), and γδ T cells identified by expression of the TCR γδ receptor.

Within the $CD4^+$ T cell population, regulatory T (Treg) cells were distinguished by high expression of CD25 combined with low or negative CD127 expression. Follicular T cells were characterized by CD185 expression. Additionally, CD4+ T cell polarization was assessed based on CD195 and CD196 expression, allowing identification of functional subsets: Th1 ($CD195^+CD196^-$), Th17 ($CD195^-CD196^+$), and Th1/17 ($CD195^+CD196^+$). CD4+ T cells negative for both markers ($CD195^-CD196^-$) represented a mix of other functional subsets, including Th0 cells.

A similar approach was used to identify $CD8^+$ T cell subpopulations. For each subset within the CD4+ and CD8+ T cells, the proportion of activated cells was determined by analyzing intermediate activation marker CD25 and late activation marker HLA-DR. Furthermore, functional compartments of T cells were evaluated for both $CD4^+$ and $CD8^+$ populations. Naïve T cells were defined by co-expression of CD45RA and CD27. Central memory (CM) T cells lacked CD45RA but retained CD27 positivity, while effector memory (EM) T cells were negative for both CD45RA and CD27. Terminal effector cells expressed CD45RA but lacked CD27.

The analysis hierarchy is illustrated in Supplementary Figure S1.

Leukocytes (CD45$^+$)

└──**Eosinophils** (CD16$^-$)

└──**Neutrophils** (CD16$^+$)

└──**Monocytes** (CD33$^+$)

| └── **Classical** (CD14$^+$/CD16$^-$)

| └── CD62L$^+$/ FcεRI $^+$

| └──CD62L$^+$/ FcεRI $^-$

| └── CD62L$^-$/ FcεRI$^+$

| └──CD62L$^-$/ FcεRI$^-$

| └──**Intermediate** (CD14$^+$/CD16$^+$)

| └── **Non-classical** (CD14$^-$/CD16$^+$)

| └── SLAN$^+$/CD36$^+$

| └──SLAN$^+$/CD36$^-$

| └── SLAN$^-$/CD36$^+$

| └──SLAN$^-$/CD36$^-$

└──**Dendritic Cells**

| └──pDC (CD303$^+$, HLA-DR$^+$)

| └── cDC1 (CD141$^{dim/-}$)

| └──CD5$^+$/CD14^{dim}

| └── CD5$^+$/CD14$^-$

| └──CD5$^-$/CD14$^-$

| └── cDC2 (CD141$^+$, CD300e$^+$)

└── **Lymphocytes (CD45^{high})**

└── **T Cells**

└──**CD4$^+$**

└── **CD8$^+$**

└── **CD4$^+$ CD8$^+$**

└── **CD4$^-$ CD8$^-$**

└── **γδ**

└── **Basophils (CD33$^+$, Slan$^+$, FcεRI$^+$, CD62L$^+$, CD14$^-$, HLA-DR$^-$, CD300e$^-$)**

CD4$^+$ T cells

└──**Treg** (CD25^{high} CD127^{low})

| └── **Treg Follicular** (CD25^{high} CD127^{low} CD185$^+$)

| └── Treg Follicular Th1 (CD25^{high} CD127^{low} CD185$^+$ CD195$^+$)

| └──Treg Follicular Th17 (CD25^{high} CD127^{low} CD185$^+$ CD196$^+$)

| └── Treg Follicular Th17/Th1 (CD25^{high} CD127^{low} CD185$^+$ CD

| └── Treg Follicular CD195-CD196- (CD25^{high} CD127^{low} CD18
CD195$^-$)

| └──**Treg Th1** (CD25^{high} CD127^{low} CD195$^+$)

| └── **Treg Th17** (CD25^{high} CD127^{low} CD196$^+$)

| └──**Treg Th17/Th1** (CD25^{high} CD127^{low} CD196$^+$ CD195$^+$)

| └── **Treg CD195-CD196-** (CD25^{high} CD127^{low} CD196$^-$ CD195$^-$)

└──**Follicular (CD185$^+$)**

| └── Follicular Th1 (CD185$^+$ CD195$^+$)

| └──Follicular Th17 (CD185$^+$ CD196$^+$)

| └── Follicular Th17/Th1 (CD185$^+$ CD196$^+$ CD195$^+$)

| └──Follicular CD195-CD196- (CD185$^+$ CD196$^-$ CD195$^-$)

└──**Th17** (CD195$^+$)

└──**Th1** (CD196$^+$)

└── **Th17/Th1** (CD195$^+$ CD196$^+$)

└──**CD195-CD196-**

Expression of CD25, HLA-DR, and memory subsets (naive, central memory, effector memory, and effector) was analyzed across all T cell subpopulations. A similar approach was used to characterize CD8$^+$ T cell subsets.

Figure S1. Detailed hierarchy of phenotypic classification of leukocytes and CD4$^+$ T cell subpopulations.

Table S2. Comparison of T cell subpopulations across maturation compartments between the control group and Luminal A group.

T cells	Maturation-associated Compartments	Control	Luminal A	*p*-value
$CD4^+$ T cells	Naïve	41 ± 13	35 ± 14	0.29
	Central Memory	45 ± 9	52 ± 11	0.04
	Effector Memory	11 ± 7.22	8.97 ± 5.40	0.46
	Effector	3.12 ± 5.86	3.40 ± 5.38	0.9
$CD8^+$ T cells	Naïve	14 ± 7.21	21 ± 18	0.51
	Central Memory	35 ± 14	35 ± 14	0.98
	Effector Memory	20 ± 14	21 ± 14	0.82
	Effector	31 ± 16	23 ± 16	0.25
$CD4^+CD8^+$ T cells	Naïve	21 ± 14	23 ± 18	0.73
	Central Memory	44 ± 21	31 ± 22	0.19
	Effector Memory	23 ± 23	17 ± 11	0.15
	Effector	12 ± 19	28 ± 30	0.19
$CD4^-CD8^-$ $\gamma\delta^-$ T cells	Naïve	18 ± 11	29 ± 15*	0.04
	Central Memory	64 ± 11	44 ± 15**	0.019
	Effector Memory	8.45 ± 4.69	8.88 ± 8.90	0.56
	Effector	9.56 ± 6.12	18 ± 11*	0.026
γδ T cells	Naïve	9.93 ± 9.67	7.66 ± 6.05	0.69
	Central Memory	50 ± 25	43 ± 28	0.43
	Effector Memory	24 ± 23	16 ± 19	0.26
	Effector	18 ± 20	34 ± 9.67	0.14

All results are expressed as mean ± SD, and statistical comparisons were performed using the Mann-Whitney non-parametric test. Statistically significant differences are indicated as $*p < 0.05$.

Table S3. Analys of principal T cell subpopulations in control and luminal A groups.

	T Cells	Controls	Luminal A	*p*-value
**CD4+**	Treg	6.54 ± 2.02	7.74 ± 2.18	0.14
	Follicular	15 ± 4.30	29 ± 17*	0.02
	Th17	9.64 ± 4.20	14 ± 6.43*	0.04
	Th1	5.89 ± 2.95	7.76 ± 6.49	0.72
	Th17/Th1	5.37 ± 8.93	2.98 ± 1.8	0.79
	CD195-CD196-	55 ± 13	40 ± 5.16*	0.02
**CD8+**	Treg	0.28 ± 0.19	0.46 ± 0,45	0.49
	Follicular	2.67 ± 1.50	17 ± 17**	0.003
	Tc17	7.55 ± 11	2.04 ± 1.64	0.09
	Tc1	32 ± 23	26 ± 21	0.44
	Tc17/Tc1	4.76 ± 3.95	2.78 ± 2.75	0.15
	CD195-CD196-	52 ± 24	52 ± 23	0.97

All results are expressed as mean ± SD, and statistical comparisons were performed using the Mann-Whitney non-parametric test. Statistically significant differences are indicated as *p < 0.05.

Table S4. Frequency of activated T cell subsets in controls and Luminal A. Activation was assessed by CD25 and HLA-DR expression in different T cell populations

	T Cells	Controls	Luminal A	*p*-value
CD4+	Treg HLA-DR$^+$	31 ± 10	34 ± 6.92	0.37
	Follicular CD25$^+$	59 ± 14	65 ± 17	0.30
	Follicular HLA-DR$^+$	5.39 ± 3.33	3.79 ± 0.98	0.11
	Th17 CD25$^+$	79 ± 14	91 ± 6.99**	0.007
	Th17 HLA-DR$^+$	4.24 ± 2.12	3.01 ± 1.03	0.21
	Th1 CD25$^+$	30 ± 21	30 ± 13	0.97
	Th1 HLA-DR$^+$	24 ± 10	26 ± 11	0.59
	Th17/Th1 CD25$^+$	65 ± 16	75 ± 12	0.05
	Th17/Th1 HLA-DR$^+$	12 ± 6.94	11 ± 5.33	0.61
	CD195$^-$CD196$^-$ CD25$^+$	41 ± 13	60 ± 15**	0.001
	CD195$^-$CD196$^-$ HLA-DR$^+$	5.33 ± 5.37	7.19 ± 3.39	0.04
CD8+	Treg HLA-DR$^+$	43 ± 16	29 ± 11*	0.02
	Follicular CD25$^+$	25 ± 32	50 ± 37	0.11
	Follicular HLA-DR$^+$	31 ± 6.88	28 ± 15	0.45
	Tc17 CD25$^+$	57 ± 30	74 ± 23	0.15
	Tc17 HLA-DR$^+$	8.53 ± 8.38	9.51 ± 7.00	0.74
	Tc1 CD25$^+$	43 ± 41	63 ± 41	0.38
	Tc1 HLA-DR$^+$	26 ± 8.89	33 ± 14	0.13
	Tc17/Tc1 CD25$^+$	45 ± 34	57 ± 36	0.40
	Tc17/Tc1 HLA-DR$^+$	14 ± 11	17 ± 18	0.63
	CD195$^-$CD196$^-$ CD25$^+$	42 ± 40	51 ± 38	0.48
	CD195$^-$CD196$^-$ HLA-DR$^+$	13 ± 9.78	16 ± 11	0.45

All results are expressed as mean ± SD, and statistical comparisons were performed using the Mann-Whitney non-parametric test. Statistically significant differences are indicated as *p < 0.05.

Table S5. Maturation compartments of CD4+ and CD8+ T cells in the control and Luminal A groups. The data show the distribution of T cell subsets, including naïve, central memory, effector memory, and terminal effector cells, across different populations such as Treg, follicular, Th17, Th1, Th17/1, and CD195$^-$CD196$^-$.

Maturation-associated	CD4$^+$ T cells	CD4	CD8$^+$ T cells	CD8

**Compartments**		Control	Luminal A	*p*-value		Control	Luminal A	*p*-value
Naïve	Treg	26 ± 10	28 ± 10	0.72	Treg	17 ± 19	15 ± 8.45	0.54
Central Memory		70 ± 10	68 ± 12	0.64		66 ± 23	65 ± 17	0.57
Effector Memory		3.74 ± 1.03	3.81 ± 1.28	0.86		12 ± 9.12	13 ± 5.10	0.49
Terminal Effector		0.30 ± 1.00	0.56 ± 1.41	0.58		4.81 ± 6.52	6.11 ± 7.02	0.50
Naïve	Follicular	22 ± 5.71	38 ± 23*	0.014	Follicular	8.30 ± 5.79	20 ± 17**	0.006
Central Memory		78 ± 6.60	60 ± 24	0.12		81 ± 14	47 ± 42**	0.008
Effector Memory		0.94 ± 0.33	0.67 ± 0.92**	0.0012		5.99 ± 7.22	2.05 ± 2.89*	0.03
Terminal Effector		0.04 ± 0,12	1.42 ± 2.85*	0.037		4.38 ± 4.63	31 ± 34**	0.009
Naïve	CD196+	9.01 ± 8.99	6.24 ± 6.28	0.35	CD196+	29 ± 23	12 ± 9.90*	0.02
Central Memory		78 ± 10	81 ± 9.06	0.33		53 ± 17	72 ± 13**	0.004
Effector Memory		13 ± 5.34	11 ± 4.93	0.47		11 ± 8.20	12 ± 7.24	0.76
Terminal Effector		0.57 ± 0.55	0.43 ± 1.03*	0.019		6.57 ± 5.21	3.39 ± 4.62	0.03
Naïve	CD195+	13 ± 16	11 ± 14	0.73	CD195+	13 ± 8.44	17 ± 11	0.26
Central Memory		36 ± 18	35 ± 15	0.72		37 ±	39 ± 22	0.76
Effector Memory		36 ± 18	44 ± 23	0.31		17 ± 12	22 ± 14	0.19
Terminal Effector		15 ± 20	9.88 ± 17	0.43		33 ± 21	22 ± 16	0.13
Naïve	$CD195^+CD196^+$	15 ± 19	7.68 ± 16*	0.033	CD195+CD196+	12 ± 9.67	18 ± 25	0.42
Central Memory		57 ± 14	62 ± 14	0.28		65 ± 20	58 ± 34	0.50
Effector Memory		25 ± 8.56	29 ± 12	0.19		12 ± 6.62	11 ± 11	0.41
Terminal Effector		3.21 ± 5.55	1.26 ± 2.10	0.20		10 ± 12	13 ± 22	0.31
Naïve		55 ± 19	38 ± 25	0.06		23 ± 15	26 ± 25	0.68

Central Memory	CD195$^-$ CD196$^-$ *CD4$^+$ T cells*	29 ± 8.32	46 ± 18**	0.0032	CD195$^-$ CD196$^-$ *CD8$^+$ T cells*	28 ± 10	31 ± 15	0.60
Effector Memory		14 ± 17	1 ± 9.22	0.64		24 ± 21	26 ± 18	0.54
Terminal Effector		2.10 ± 2.62	3.20 ± 8.85	0.35		25 ± 22	17 ± 14	0.31

All results are expressed as mean ± SD, and statistical comparisons were performed using the Mann-Whitney non-parametric test. Statistically significant differences are indicated as *p < 0.05.

Table S6. Phenotypic characterization of CD4$^+$ and CD8$^+$ T cell subsets in peripheral blood from healthy controls and luminal A breast cancer patients.

		**T Cells**	**Controls**	**Luminal A**	***p*-value**
**CD4+**	**Treg**	Treg Follicular	28 ± 7.6	26 ± 10	0.59
		Treg Follicular Th17	13 ± 6.32	21 ± 12*	0.03
		Treg Follicular Th1	12 ± 12	8.00 ± 6.83	0.35
		Treg Follicular Th17/Th1	9.91 ± 12	5.93 ± 4.61	0.28
		Treg Follicular CD195-CD196-	65 ± 21	66 ± 21	0.90
		Treg Th17	11 ± 5.59	22.4 ± 15.79*	0.01
		Treg Th1	11 ± 6.23	7.30 ± 4.94	0.15
		Treg Th17/Th1	11 ± 5.51	14.3 ± 7.57	0.22
		Treg CD195-CD196-	40 ± 15	31 ± 11	0.12
	**Follicular**	Follicular Th17	21 ± 19	25 ± 13	0.30
		Follicular Th1	4.99 ± 5.96	9.49 ± 15	0.58
		Follicular Th17/Th1	4.51 ± 3.65	2.81 ± 5.31*	0.026
		Follicular CD195-CD196-	70 ± 20	63 ± 18	0.24
**CD8+**	**Treg**	Treg Follicular	22 ± 26	27 ± 20	0.53
		Treg Tc17	14 ± 7.78	16 ± 11	0.83
		Treg Tc1	24 ± 14	14 ± 3.95	0.26
		Treg Tc17/Tc1	11 ± 9.74	5.6 ± 0.81	0.83
		Treg CD195-CD196-	29 ± 18	40 ± 13	0.52
	**Follicular**	Follicular Tc17	11 ± 11	3.29 ± 3.49*	0.018
		Follicular Tc1	39 ± 30	21 ± 30	0.13
		Follicular Tc17/Tc1	21 ± 25	2.59 ± 3.40*	0.013
		Follicular CD195-CD196-	28 ± 19	74 ± 31***	0.0001

All results are expressed as mean ± SD, and statistical comparisons were performed using the Mann-Whitney non-parametric test. Statistically significant differences are indicated as $*p < 0.05$.

Table S7. Activation profile of $CD4^+$ and $CD8^+$ T cell subsets in peripheral blood from healthy controls and luminal A breast cancer patients.

		**T Cells**	**Controls**	**Luminal A**	***p*-value**
**CD4+**	**Treg**	Treg Follicular HLA-DR$^+$	8.21 ± 4.98	12 ± 7.59	0.26
		Treg Follicular Th17 HLA-DR$^+$	10 ± 4.19	8.17 ± 3.19	0.16
		Treg Follicular Th1 HLA-DR$^+$	19 ± 9.43	8.17 ± 3.20	0.05
		Treg Follicular Th17/Th1 HLA$^-$DR$^+$	17 ± 7.63	18 ± 7.35	0.86
		Treg Follicular CD195$^-$CD196$^-$ HLA-DR$^+$	5.93 ± 4.99	2.44 ± 1.26*	0.02
		Treg Th17 HLA-DR$^+$	41 ± 10	40 ± 6.32	0.82
		Treg Th1 HLA-DR$^+$	50 ± 14	51 ± 11	0.95
		Treg Th17/Th1 HLA-DR$^+$	48 ± 14	57 ± 9.47	0.06
		Treg CD195$^-$CD196$^-$ HLA-DR$^+$	31 ± 13	37 ± 11	0.25
	**Follicular**	Follicular Th17 CD25$^+$	67 ± 14	73 ± 30	0.50
		Follicular Th17 HLA-DR$^+$	5.23 ± 4.28	2.3 ± 1.02*	0.02
		Follicular Th1 CD25$^+$	35 ± 23	49 ± 32	0.18
		Follicular Th1 HLA-DR$^+$	21 ± 12	21 ± 14	0.99
		Follicular Th17/Th1 CD25$^+$	60 ± 15	68 ± 20	0.24
		Follicular Th17/Th1 HLA-DR$^+$	15 ± 12	9.97 ± 5.79	0.18
		Follicular CD195$^-$CD196$^-$ CD25$^+$	63 ± 12	64 ± 22	0.84
		Follicular CD195$^-$CD196$^-$ HLA-DR$^+$	3.63 ± 2.25	2.71 ± 1.37	0.22
**CD8+**	**Treg**	Treg Follicular HLA-DR$^+$	40 ± 31	33 ± 20	0.61
		Treg Tc17 HLA-DR$^+$	34 ± 28	22 ± 14	0.49
		Treg Tc1 HLA-DR$^+$	43 ± 12	63 ± 6.17*	0.03
		Treg Tc17/Tc1 HLA-DR$^+$	36 ± 19	39 ± 18	0.81
		Treg CD195-CD196- HLA-DR$^+$	16 ± 9.47	24 ± 7.25	0.22
	**Follicular**	Follicular Tc17 CD25$^+$	51 ± 31	54 ± 23	0.84
		Follicular Tc17 HLA-DR$^+$	25 ± 24	21 ± 21	0.67
		Follicular Tc1 CD25$^+$	37 ± 40	54 ± 23	0.43
		Follicular Tc1 HLA-DR$^+$	35 ± 10	49 ± 32	0.37
		Follicular Tc17/Tc1 CD25$^+$	36 ± 27	43 ± 28	0.57
		Follicular Tc17/Tc1 HLA-DR$^+$	44 ± 21	60 ± 32	0.16
		Follicular CD195$^-$CD196$^-$ CD25$^+$	44 ± 36	58 ± 34	0.34
		Follicular CD195$^-$CD196$^-$ HLA-DR$^+$	23 ± 14	25 ± 13	0.77

All results are expressed as mean ± SD, and statistical comparisons were performed using the Mann-Whitney non-parametric test. Statistically significant differences are indicated as *p < 0.05.

Table S8. Maturation-associated distribution of follicular $CD4^+$ and $CD8^+$ T cell subsets in peripheral blood from healthy controls and luminal A breast cancer patients.

**Maturation-associated Compartments**	**$CD4^+$ T cells**	**CD4**			**$CD8^+$ T cells**	**CD8**		
		Control	Luminal A	*p*-value		Control	Luminal A	*p*-value
Naïve	Follicular $CD195^+$	15 ± 17	39 ± 38*	0.03	Follicular $CD195^+$	5.68 ± 3.87	13 ± 13	0.05
Central Memory		82 ± 17	53 ± 40*	0.02		87 ± 9.85	46 ± 45**	0.004
Effector Memory		2.95 ± 2.29	1.41 ± 1.08*	0.04		4.71 ± 8.04	2.28 ± 4.11	0.34
Terminal Effector		0.14 ± 0.35	5.43 ± 14	0.15		2.66 ± 5.04	39 ± 40**	0.003
Naïve	Follicular $CD196^+$	14 ± 9.37	22 ± 25	0.24	Follicular $CD196^+$	16 ± 19	32 ± 19	0.05
Central Memory		86 ± 7.03	76 ± 25	0.18		72 ± 23	44 ± 26*	0.007
Effector Memory		2.16 ± 3.25	0.94 ± 0.88	0.21		5.92 ± 5.08	1.83 ± 3.77*	0.03
Terminal Effector		0.00 ± 0.00	0.25 ± 0.52	0.08		4.78 ± 6.25	22 ± 18**	0.0024
Naïve	Follicular $CD195^+$ $CD196^+$	14 ± 9.1	33 ± 30*	0.04	Follicular $CD195^+$ $CD196^+$	19 ± 18	22 ± 20	0.72
Central Memory		79 ± 8.64	60 ± 28*	0.03		64 ± 29	30 ± 41*	0.02
Effector Memory		6.52 ± 6.63	5.00 ± 5.04	0.51		3.55 ± 3.57	5.26 ± 13	0.65
Terminal Effector		0.009 ± 0.03	1.78 ± 2.84*	0.03		13.93 ± 16	43.48 ± 34.49**	0.009
Naïve	Follicular $CD195^-$ $CD196^-$	25 ± 8.68	47 ± 24**	0.005	Follicular $CD195^-$ $CD196^-$	16 ± 10	23 ± 18	0.20
Central Memory		73 ± 8.09	51 ± 25**	0.005		75 ± 9.34	46 ± 41*	0.02
Effector Memory		0.83 ± 0.43	0.41 ± 0.17**	0.003		8.38 ± 11	1.90 ± 3.26	0.05
Terminal Effector		0.009 ± 0.02	1.69 ± 2.86*	0.03		1.23 ± 1.48	29 ± 34**	0.007

All results are expressed as mean ± SD, and statistical comparisons were performed using the Mann-Whitney non-parametric test. Statistically significant differences are indicated as *p < 0.05.

Table S9. Phenotypic and activation profiling of $CD4^{+}CD8^{+}$, $CD4^{-}CD8^{-}$ and γδ T cell subsets in peripheral blood from healthy controls and luminal A breast cancer patients.

	T Cells	Controls	Luminal A	*p*-value
**CD4+CD8+**	Treg	1.06 ± 0.92	1.53 ± 1.46	0.34
	Treg HLA-DR+	39 ± 25	19 ± 18*	0.03
	Follicular	5.59 ± 3.71	23 ± 26*	0.02
	Follicular $CD25^{+}$	51 ± 26	55 ± 29	0.68
	Follicular HLA-DR^{+}	23 ± 12	22 ± 15	0.86
	$CD196^{+}$	9.08 ± 5.46	8.39 ± 12*	0.04
	$CD196^{+}$ CD25+	54 ± 19	71 ± 15*	0.018
	$CD196^{+}$ HLA-DR^{+}	8.63 ± 6.32	8.70 ± 6.77	0.97
	$CD195^{+}$	23 ± 20	6.59 ± 6.58	0.84
	$CD195^{+}$ CD25+	35 ± 29	47 ± 26	0.27
	$CD195^{+}$ HLA-DR^{+}	27 ± 11	22 ± 10	0.24
	$CD196^{+}$ $CD195^{+}$	6.82 ± 4.45	3.68 ± 3.02*	0.01
	$CD196^{+}CD195^{+}$ CD25+	40 ± 21	49 ± 19	0.29
	$CD196^{+}CD195^{+}$ HLA-DR^{+}	19 ± 14	29 ± 22	0.18
	$CD195^{-}CD196^{-}$	55 ± 17	57 ± 28	0.84
	$CD195^{-}CD196^{-}$ $CD25^{+}$	43 ± 31	49 ± 31	0.67
	$CD195^{-}CD196^{-}$ HLA-DR^{+}	14 ± 12	13 ± 12	0.93
**CD4-CD8-**	Treg	1.03 ± 1.23	2.36 ± 2.09	0.05
	Treg HLA-DR+	49 ± 40	77 ± 13	0.08
	Follicular	17 ± 12	28 ± 22	0.12
	Follicular $CD25^{+}$	24 ± 13	29 ± 19	0.42
	Follicular HLA-DR^{+}	39 ± 30	53 ± 21	0.18
	$CD196^{+}$	9.98 ± 9.08	7.51 ± 9.06	0.49
	$CD196^{+}$ CD25+	27 ± 14	57 ± 17***	0.001
	$CD196^{+}$ HLA-DR^{+}	34 ± 24	28 ± 25	0.58
	$CD195^{+}$	21 ± 13	17 ± 8.89	0.45
	$CD195^{+}$ CD25+	11 ± 15	24 ± 23	0.12
	$CD195^{+}$ HLA-DR^{+}	38 ± 19	41 ± 18	0.69
	$CD196^{+}$ / $CD195^{+}$	28 ± 19	19 ± 14	0.21
	$CD196^{+}/CD195^{+}$ CD25+	23 ± 15	46 ± 27*	0.02
	$CD196^{+}/CD195^{+}$ HLA-DR^{+}	28 ± 29	24 ± 33	0.77
	$CD195^{-}CD196^{-}$	23 ± 17	26 ± 20	0.58
	$CD195^{-}CD196^{-}$ $CD25^{+}$	13 ± 8.21	42 ± 28**	0.002
	$CD195^{-}CD196^{-}$ HLA-DR^{+}	24 ± 15	25 ± 19	0.83

	Treg	0.07 ± 0.19	0.29 ± 0.58	0.21
	Treg HLA-DR+	29 ± 34	59 ± 34	0.21
	Follicular	2.21 ± 2.34	21 ± 27*	0.02
	Follicular CD25$^+$	17 ± 15	18 ± 9.43	0.92
	Follicular HLA-DR$^+$	23 ± 20	24 ± 18	0.83
	CD196$^+$	7.83 ± 12	6.35 ± 7.85	0.71
	CD196$^+$ CD25+		38 ± 27	0.39
	CD196$^+$ HLA-DR$^+$	18 ± 14	16 ± 13	0.67
**γδ**	CD195$^+$	41 ± 27	28 ± 25	0.19
	CD195$^+$ CD25+	21 ± 18	31 ± 15	0.13
	CD195$^+$ HLA-DR$^+$	25 ± 19	26 ± 23	0.92
	CD196$^+$ / CD195$^+$	15 ± 14	13 ± 19	0.71
	CD196$^+$/CD195$^+$ CD25+	37 ± 17	44 ± 20	0.32
	CD196$^+$/CD195$^+$ HLA-DR$^+$	20 ± 13	21 ± 19	0.81
	CD195$^-$CD196$^-$	33 ± 29	32 ± 31	0.94
	CD195$^-$CD196$^-$ CD25$^+$	11 ± 8.58	18 ± 14	0.12
	CD195$^-$CD196$^-$ HLA-DR$^+$	17 ± 16	19 ± 14	0.75

All results are expressed as mean ± SD, and statistical comparisons were performed using the Mann-Whitney non-parametric test. Statistically significant differences are indicated as *$p < 0.05$.
